# Coinfection with infectious bronchitis virus exacerbates the pathogenicity of *Riemerella anatipestifer* in chickens

**DOI:** 10.3389/fvets.2026.1788133

**Published:** 2026-03-17

**Authors:** Yang Cong, Hui Chen, Yuehua Gao, Xiuli Ma, Xiaofei Song, Xiaodong Liu, Yufeng Li, Zhuoming Qin, Junfeng Lv

**Affiliations:** 1 Poultry Institute, Shandong Academy of Agricultural Sciences, Jinan, China; 2 College of Veterinary Medicine, Qingdao Agricultural University, Qingdao, China; 3 Qilu Animal Health Products Co., Ltd., Jinan, China; 4 Shandong Provincial Key Laboratory of Livestock and Poultry Breeding, Qingdao, China

**Keywords:** chickens, coinfection, IBV, increased pathogenicity, *Riemerella anatipestifer*

## Abstract

**Introduction:**

Since its initial isolation from chickens, *Riemerella anatipestifer* has emerged as an increasingly prevalent pathogen in major poultry-producing regions, causing substantial economic losses, particularly through reduced egg production.

**Methods:**

In the present study, an epidemiological investigation was conducted to detect coinfected pathogens in *R. anatipestifer*-positive clinical samples. Based on the epidemiological findings, the impact of coinfection with infectious bronchitis virus (IBV) on the pathogenicity of *R. anatipestifer* was evaluated in specific-pathogen-free (SPF) chickens.

**Results:**

Epidemiological analysis revealed that IBV was the most frequently detected coinfecting pathogen (11.43%) in *R. anatipestifer*-positive samples. Animal challenge experiments demonstrated that bacterial loads in the liver, spleen, and brain were significantly higher in coinfected chickens than in those infected with *R. anatipestifer* alone. Notably, the incidence of oviduct obstruction was markedly elevated in the coinfected group (100%) compared to the group infected solely with *R. anatipestifer* (40%).

**Discussion:**

These results suggested that IBV coinfection exacerbated the pathogenicity of *R. anatipestifer* in chickens. These findings highlight the critical role of polymicrobial interactions in modulating bacterial virulence and provide a foundation for developing integrated control strategies against *R. anatipestifer*.

## 1 Introduction

*Riemerella anatipestifer* (*R. anatipestifer*), a member of the family *Weeksellaceae* within the order *Flavobacteriale*, is characterized as a Gram-negative, non-motile, non-spore-forming rod (1, 2). As a major pathogen that primarily affects ducks and geese, it causes acute or chronic septicemia, leading to substantial economic losses in the poultry industry worldwide, particularly in China (3–7). The bacterium has at least 25 distinct serotypes with minimal cross-agglutination, posing significant challenges for broad-spectrum vaccine development (8–10). Therefore, a thorough understanding of the epidemiology and pathogenesis of *R. anatipestifer* is essential for the development of effective disease control strategies.

In recent years, *R. anatipestifer* has been identified as the causative agent of clinical signs such as reduced egg production, decreased hatching rate, and lameness in chickens (11, 12). Since its initial isolation from hens in 2019, epidemiological surveillance has indicated that the bacterium has become increasingly prevalent, with rapid geographical expansion and a broad host range across various chicken types, and emerged as a significant concern for the chicken industry (6, 13). Mirroring the diversity observed in ducks, multiple serotypes including serotypes 1, 2, 4, 6, 7, and 10 have been detected in chickens, several of which had been confirmed to induce typical clinical diseases in hens (6, 12, 14, 15). Notably, the pathogenicity of *R. anatipestifer* in chickens has demonstrably evolved since its first isolation. Studies using strains isolated earlier reported only mild or subclinical infections in challenge experiments (11, 16). In contrast, recent animal challenge studies with contemporary isolates have described severe pathological manifestations such as oviductal distension, follicular regression, yolk collapse and discoloration, peritoneal effusion, and atrophy of the uterine and isthmus segments of the oviduct (17). The molecular mechanisms underlying this apparent increase in pathogenicity need to be elucidated in future studies.

The epidemiology of *R. anatipestifer* infection is influenced by a complex interplay of factors. Although some studies have reported no distinct seasonal pattern of the disease, peaks in incidence were frequently associated with specific environmental stressors (7). In chicken populations, a higher frequency of cases has been consistently observed between September and December, which correlates with specific seasonal conditions (6). Furthermore, management-related factors such as poor sanitation, inadequate nutrition, and concurrent infections with other pathogens were known to significantly increase susceptibility and outbreak severity in poultry flocks (18). Hence, a clear understanding of these contributing factors is essential for the development of effective disease control and prevention strategies.

Infectious bronchitis virus (IBV), a member of the genus *Gammacoronavirus* within the family *Coronaviridae*, is the causative agent of infectious bronchitis (IB) and has emerged as a critical threat to the poultry industry in China (19, 20). Based on the sequence of the spike 1 (S1) structural gene, IBV was classified into nine genotypes (GI–GIX), with genotype GI further subdivided into 29 distinct lineages (21, 22). Epidemiological investigations conducted in recent years have revealed that GI-19 was the most frequently isolated genotype in China, accounting for over 70% of cases, followed by GI-13 (20%) and GI-22 (4%) (23, 24). Due to the widespread use of live-attenuated vaccines, GI-19 strains had undergone continuous evolution driven by immune pressure and genetic mutations, resulting in the emergence of novel variants with altered pathogenicity and molecular characteristics (20, 25, 26).

In the present study, a thorough analysis of coinfection prevalence in *R. anatipestifer*-positive samples revealed that IBV was the most frequent coinfection. The effect of IBV coinfection on *R. anatipestifer* pathogenicity was evaluated in SPF chickens. Collectively, our findings advanced the understanding of polymicrobial interactions in avian pathology and offer a framework for devising integrated control strategies against *R. anatipestifer*.

## 2 Materials and methods

### 2.1 Ethical statement

Animal husbandry and experimental protocols adhered strictly to the Guidelines for the Care and Use of Laboratory Animals established by the Poultry Institute of the Shandong Academy of Agricultural Sciences (SAAS-2025-S035).

### 2.2 Animals and strains

SPF chickens and SPF chicken eggs were purchased from Shandong Haotai Laboratory Animal Breeding Co., Ltd. (Jinan, China). SPF chickens were individually housed in isolators under controlled temperature conditions with ad libitum access to feed and water. *R. anatipestifer* serotype 10 strain (NMDC access no. NMDCN0009F7A) was isolated from hens and preserved in our laboratory (12). *R. anatipestifer* colonies were grown on tryptic soy agar (TSA) supplemented with 4% fetal bovine serum (FBS) (Thermo Fisher, Shanghai, China) at 37 °C under anaerobic conditions for 24 h, followed by culture in tryptic soy broth (TSB). Bacteria were cultured and counted prior to animal experiment. IBV strain GI-19 (NMDC access no. NMDCN000A68N) was isolated from chickens and preserved in our laboratory (24). A viral stock was prepared from 9-day-old SPF embryonated chicken eggs, and the viral titer was measured using 9-day-old SPF chicken eggs and expressed as EID_50_/mL.

### 2.3 Detection of coinfected pathogens in *Riemerella anatipestifer*-positive clinical samples

*Riemerella anatipestifer*-positive samples were collected between May 2024 and April 2025. Total DNA/RNA were extracted from homogenized tissues using the VAMNE Magnetic Pathogen DNA/RNA Kit (Vazyme, Nanjing, China), and the DNA/RNA concentration and purity were assessed spectrophotometrically using a NanoDrop One system (Thermo Fisher). DNA pathogens were detected using 2 × Rapid Taq Master Mix (Vazyme), whereas RNA pathogens were detected using an AccurSTART One Step RT-PCR Kit (Vazyme). Both assays were conducted according to the manufacturer’s instructions, with an annealing temperature of 55 °C. Primers specific to *Escherichia coli*, *Salmonella enterica*, *Avibacterium paragallinarum*, IBV, infectious bursal disease virus (IBDV), chicken infectious anemia virus (CIAV), and fowl adenovirus serotype (FAdV) were designed and listed in Table 1. PCR products were analyzed using gel electrophoresis.

**Table 1:** Primers used in this study.

Name	Sequence (5′—3′)	Product (bp)
*E. coli* ^a^	F: AAGGCGATTCAGCCACGACG	860
	R: CAGCGACGCAAGATAACG	
*S. enterica* ^b^	F: CTTTGGTCGTAAAATAAGGCG	260
	R: TGCCCAAAGCAGAGAGATTC	
*A. paragallinarum* ^c^	F: CCTGCCCCATACGCTGTTCAAC	193
	R: ATAGCTTGCTCTACCGCACAAT	
IBV	F: CCTGGAAACGAACGGTAGACC	142
	R: TAGTGGGCGTCCTA GTGCTG	
IBDV	F: TGGGAACGTCCTAGTAGG	700
	R: ATCGGCCGTATTCTGTGAC	
CIAV	F: CGCGCAGGGGCAAGTAATTT	269
	R: CTTGGGTTGATCGGTCCTCA	
FAdV^d^	F: GTGGTRTCCATGTTGGT	494
	R: ATGTACGCYTCCGCCCTC	

^a,b,c^ Universal primers for *E. coli*, *S. enterica*, and *A. paragallinarum*, respectively. ^d^ Primers for FAdV group I.

### 2.4 Animal experiments

Seventy 28-day-old SPF female chickens were randomly allocated to four groups and housed in separate isolation units. Group 1 (*n* = 20) was subcutaneously inoculated with 0.5 mL of *R. anatipestifer* bacterial suspension (1 × 10^10^ colony-forming units, CFU) on day 4. Group 2 (*n* = 20) received a subcutaneous injection of 0.5 mL of IBV suspension (10^5^ EID₅₀) on day 1. Group 3 (*n* = 20) was inoculated with IBV on day 1, followed by *R. anatipestifer* on day 4, using the same doses and routes as the respective single-infection groups. The control group (*n* = 10) received an equivalent volume of PBS via the same route on day 1. All chickens were monitored for 14 days. To calculate morbidity, 10 chickens from each infected group were marked individually. On days 7, 10, and 13, three chickens from each group were weighed and euthanized by immediate exsanguination following mechanical stunning for the collection of liver, spleen, and brain tissues. At the end of the experiment, all chickens were euthanized and subjected to postmortem examination to evaluate anatomical lesions.

### 2.5 Detection of bacterial loads

Bacterial loads in the liver, spleen, and brain tissues of infected chickens were quantified. Tissue samples (0.1 g liver, 0.05 g spleen, 0.1 g brain) were homogenized in 1 mL of TSB using a mechanical grinder. The homogenates were serially diluted ten-fold in TSB, plated on TSA, and incubated under anaerobic conditions at 37 °C for 24 h. For each time point, three tissue specimens were examined, with each specimen subjected to three independent counts. Bacterial colonies were counted and expressed as CFU per gram (CFU/g) of tissue.

### 2.6 Statistical analysis

Data were analyzed using the Student’s *t*-test or a one-way ANOVA using Prism statistical software (GraphPad, United States). The data were expressed as mean values ± SD. Statistical significance of data was considered at *p* < 0.05.

## 3 Results

### 3.1 Epidemiology of coinfected pathogens in *Riemerella anatipestifer* positive samples

**Figure 1:**
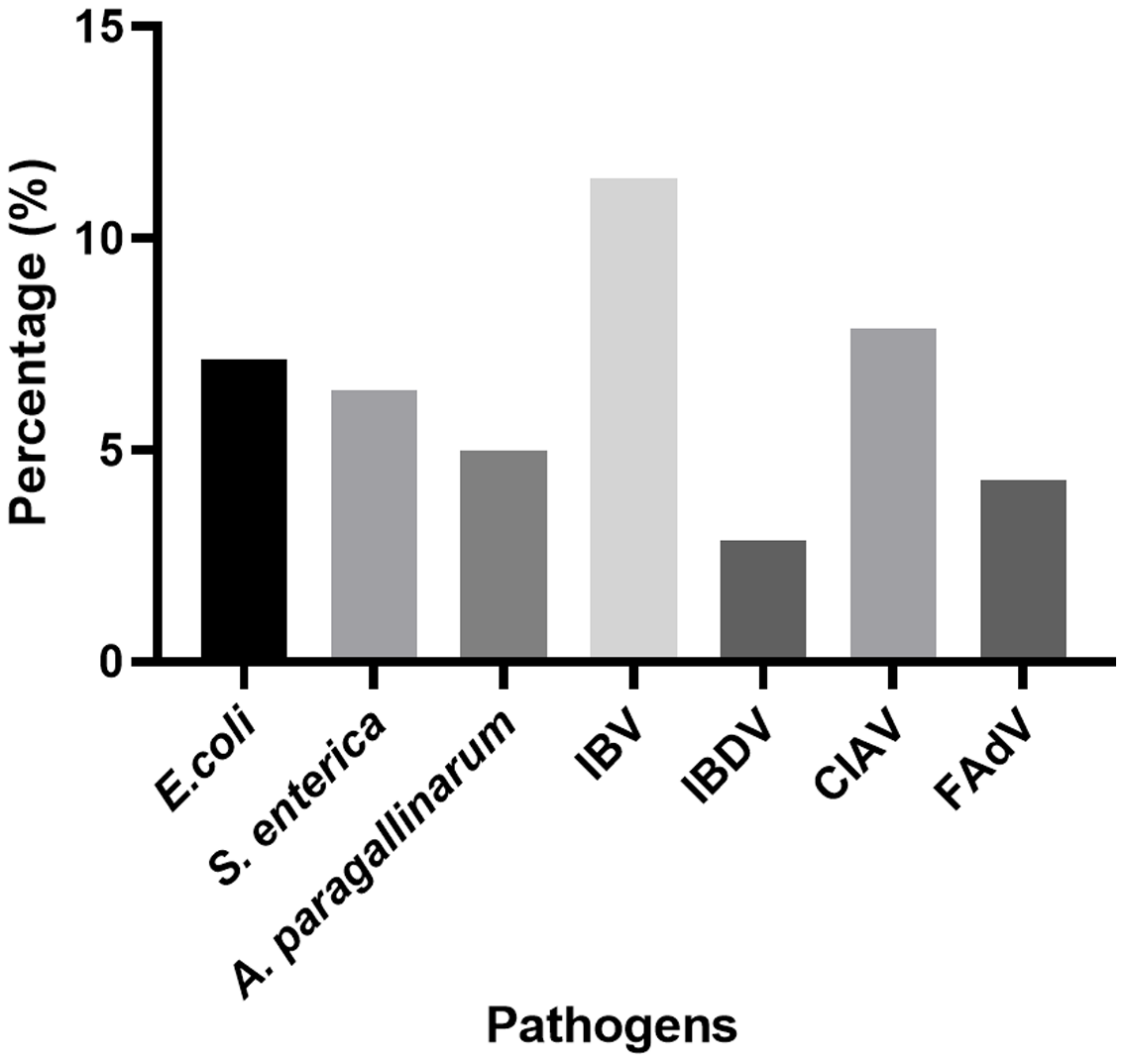
Coinfection prevalence of *R. anatipestifer*-positive samples. The epidemiology was determined using 140 clinical samples collected between May 2024 and April 2025.

Between May 2024 and April 2025, 140 *R. anatipestifer*-positive clinical samples were collected in our laboratory (Supplementary Table S1) (15). Screening for coinfected pathogens revealed IBV as the most frequently detected agent (16/140, 11.43%), followed by CIAV (11/140, 7.86%), *E. coli* (10/140, 7.14%), *S. enterica* (9/140, 6.43%), *A. paragallinarum* (7/140, 5.00%), FAdV (6/140, 4.29%), and IBDV (4/140, 2.86%) (Figure 1).

### 3.2 Animal experiment

No mortality was recorded during observation period. In contrast to chickens in the control group, which displayed no clinical signs, all chickens in the infection groups developed lethargy and reduced feed intake. Furthermore, respiratory signs were present in the IBV-challenged group.

**Figure 2:**
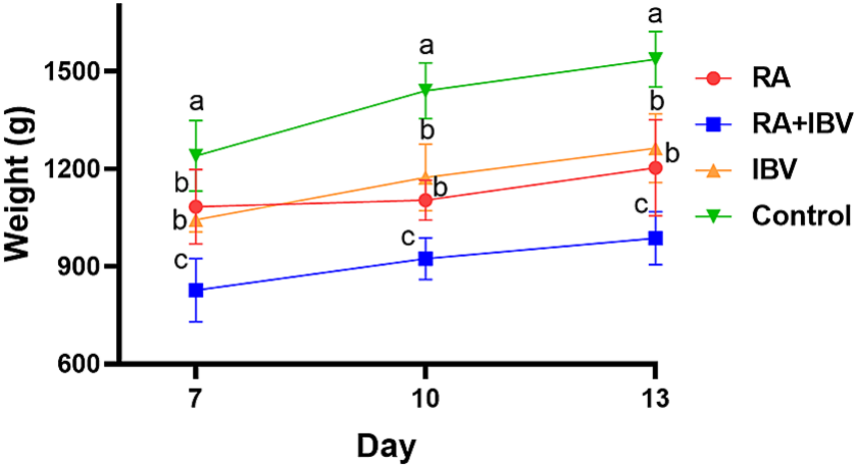
Body weight of chickens in different groups. Three chickens from each group were weighed at each timepoint. RA indicates *R. anatipestifer*. Data were expressed as mean ± SD. Different letters indicate *p* < 0.005.

The body weight of chickens in the control group increased from 1,240 g on day 7 to 1,537 g on day 13, significantly higher than that of chickens in all infected groups (*p* < 0.005) (Figure 2). No significant differences in body weight were observed between the groups infected with *R. anatipestifer* or IBV alone. In contrast, chickens in the coinfected group exhibited markedly lower body weights at all time points (827 g on day 7, 923 g on day 10, and 987 g on day 13), which were significantly lower than those in chickens in both single-infection groups (*p* < 0.005) (Figure 2).

**Figure 3:**
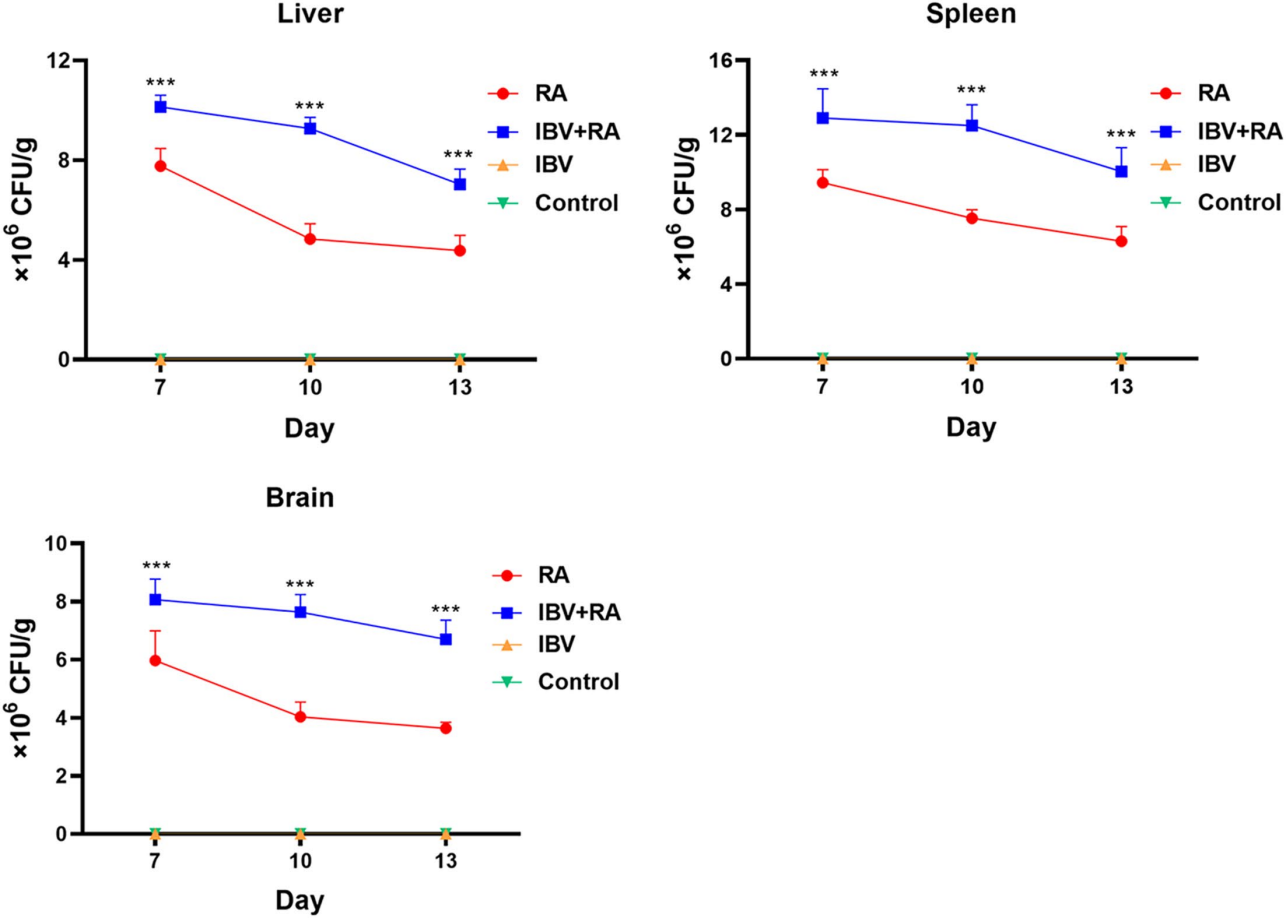
Bacterial loads in chicken tissues of different groups. RA indicates *R. anatipestifer*. Tissues of liver, spleen, and brain were collected and homogenized, and bacterial colonies grown on TSA plates were counted. Three chickens in each time point were used, and data were expressed as mean ± SD (***, *p* < 0.005).

Bacterial loads in the liver, spleen, and brain were quantified for all groups, and no bacteria were isolated from the chickens in the IBV-infected or control groups. Bacterial counts in the liver of chickens in the coinfected group were significantly higher (*p* < 0.005) than those of chickens in the group infected with *R. anatipestifer* alone at all time points, measuring 10.1 × 10^6^, 9.3 × 10^6^, and 7.0 × 10^6^ CFU/g on days 7, 10, and 13, respectively (Figure 3). Similarly, significantly higher bacterial loads (*p* < 0.005) were observed in the spleens of chickens in the coinfected groups (12.9 × 10^6^, 12.5 × 10^6^, and 10.0 × 10^6^ CFU/g on days 7, 10, and 13) than in those of chickens in the group infected with *R. anatipestifer* alone (9.4 × 10^6^, 7.5 × 10^6^, and 6.3 × 10^6^ CFU/g on the corresponding days) (Figure 3). This trend was also evident in brain tissue, where bacterial loads in the coinfected group (8.1 × 10^6^, 7.6 × 10^6^, and 6.7 × 10^6^ CFU/g) consistently exceeded those in the single-infection group (6.0 × 10^6^, 4.0 × 10^6^, and 3.6 × 10^6^ CFU/g on days 7, 10, and 13), with all differences being statistically significant (*p* < 0.005) (Figure 3).

**Figure 4:**
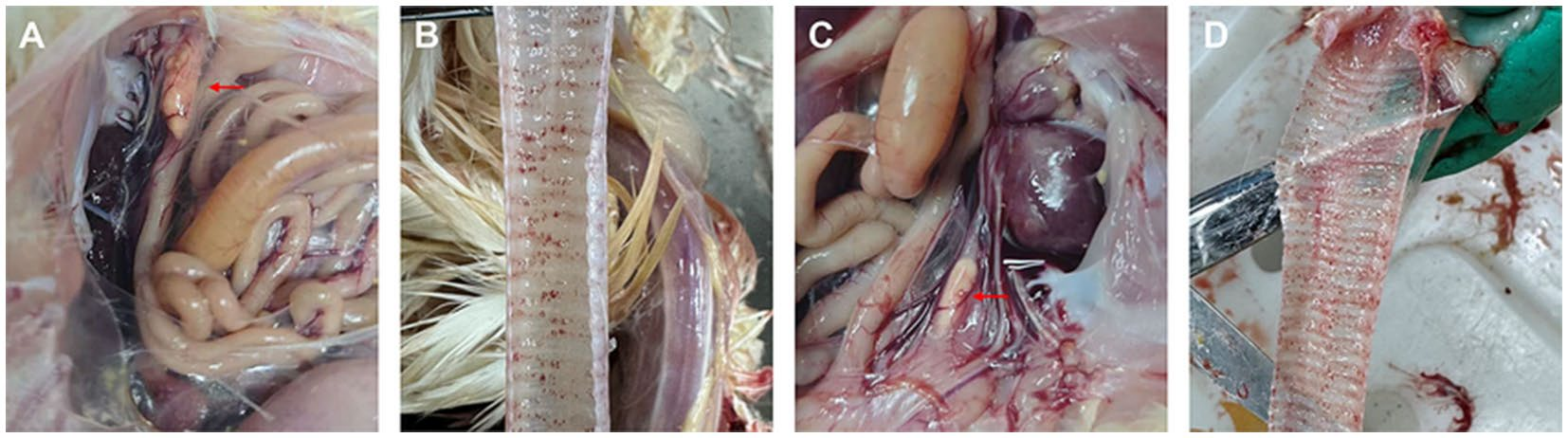
Pathological manifestation in chickens in infected groups. **(A)** Oviduct obstruction in *R. anatipestifer* infected chicken. **(B)** Tracheal hemorrhages in IBV infected chicken. **(C)** Oviduct obstruction in coinfected chicken. **(D)** Tracheal hemorrhages in coinfected chicken. Red arrows indicate sites of oviduct obstruction.

**Figure 5:**
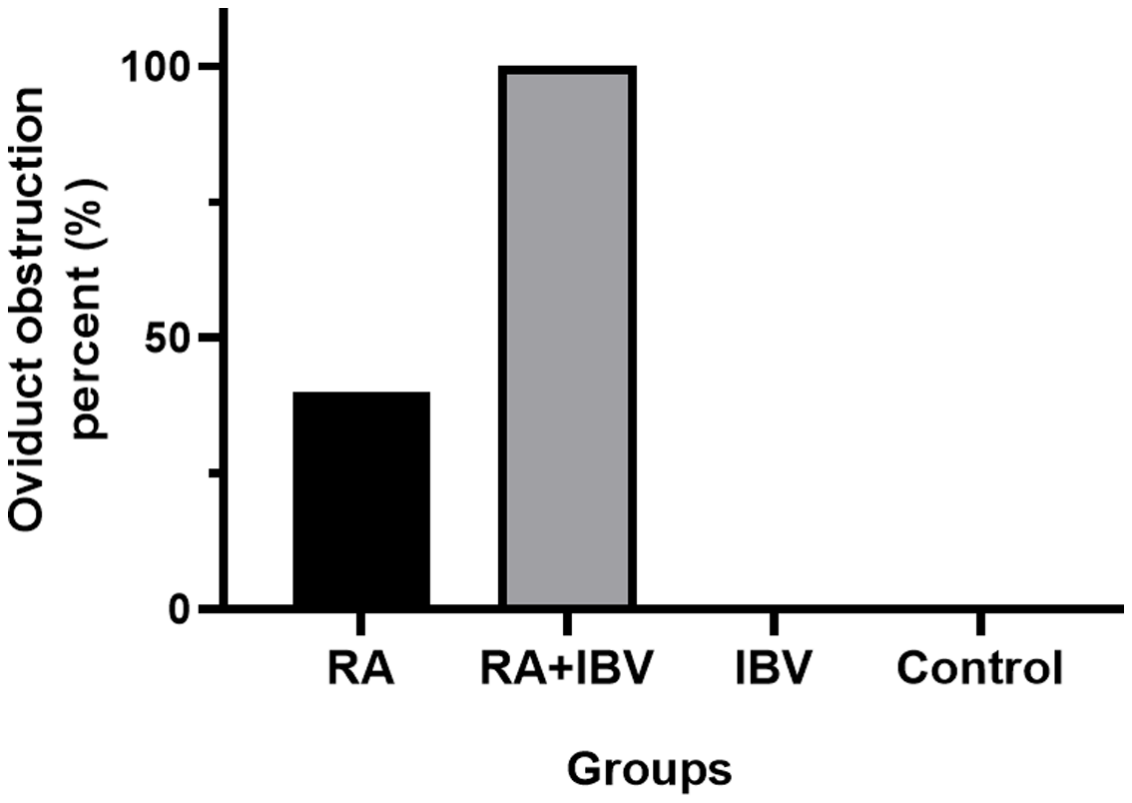
Incidence of oviduct obstruction in different groups. Chickens were euthanized and subjected to postmortem examination to evaluate anatomical lesions on day 14. RA indicates *R. anatipestifer*.

Postmortem examination revealed no gross anatomical lesions in control chickens. Chickens infected with *R. anatipestifer* and those coinfected with *R. anatipestifer* and IBV presented with oviduct obstruction (Figure 4). The incidence of oviduct obstruction was markedly higher in the coinfected group (100%) than in the group infected with *R. anatipestifer* alone (40%) (Figure 5). All birds challenged with IBV exhibited depressing, coughing during the experiment, and tracheal hemorrhages appeared upon necropsy (Figure 4).

## 4 Discussion

Since its initial isolation from chickens, the pathogenicity of *R. anatipestifer* has markedly increased. Infections have evolved from causing mild or subclinical symptoms in certain locations to inducing severe pathological manifestations across most major poultry-producing regions (6, 11, 15, 16). This enhancement in virulence is likely driven, in part, by bacterial evolution, although other factors, including environmental conditions, host status, farm management, and concurrent infections with other pathogens, might also be contributing factors. Screening of *R. anatipestifer*-positive clinical samples revealed coinfection with a range of other pathogens, most notably IBV, which was detected at a rate of 11.43%. Accordingly, we specifically evaluated the influence of coinfection with IBV on the pathogenicity of *R. anatipestifer*.

As a highly transmissible respiratory pathogen in poultry, IBV causes respiratory distress, reduced weight gain, compromised egg quality, and decreases production (27–29). Coinfections of respiratory agents including IBV, Newcastle disease virus (NDV), avian influenza virus (AIV), and *Mycoplasma gallisepticum* (MG) with other pathogens are common in chickens, as chickens infected with these pathogens showed a decline in immune protection (30–32). Coinfection of IBV with AIV (H9N2) in SPF chickens enhances H9N2 replication, alters telomerase activity, and disrupts cellular metabolism, thereby exacerbating disease severity and mortality (33, 34). Similarly, concurrent infection with bacterial pathogens such as *E. coli* results in more severe clinical manifestations than single infections, providing a useful model for pathogenesis studies (35). Given the widespread prevalence of *R. anatipestifer* in chicken populations, a high coinfection rate with IBV was epidemiologically plausible and was a focus of this investigation.

Laying hens infected with *R. anatipestifer* develop oviduct obstruction, which is the primary cause of reduced egg production. Previous studies reported that 30-, 60-, and 90-day-old hens challenged with *R. anatipestifer* serotypes 1, 5, or 10 developed oviduct obstruction at rates ranging from 22.2 to 66.7% (12). Consistent with these findings, we observed an obstruction rate of 40% in the group infected with *R. anatipestifer* alone. However, coinfection with IBV dramatically increased this rate to 100%, indicating that IBV exacerbates the oviduct pathology induced by *R. anatipestifer*. IBV is an established cause of salpingitis that could directly infect the oviduct and inducing lesions (36). Although no overt oviduct lesions were detected in chickens infected with IBV alone in the present study, the mechanism by which IBV promoted obstruction required further investigation. Notably, bacterial loads in the tissues of chickens infected with *R. anatipestifer* alone were significantly lower than those in the tissues of coinfected chickens. As *R. anatipestifer* infection typically occurs via the respiratory tract, respiratory lesions induced by IBV might facilitate bacterial invasion. Furthermore, IBV-induced immunosuppression could compromise the host’s capacity to clear *R. anatipestifer*. Collectively, these mechanisms may account for the increased severity of oviduct obstruction observed in coinfected hens.

## 5 Conclusion

In conclusion, the analysis of *R. anatipestifer*-positive samples revealed that IBV was the most frequent coinfection among those tested pathogens. In SPF chickens, IBV coinfection facilitated *R. anatipestifer* infection, compromised bacterial clearance by the host, and consequently led to a significantly increased incidence of oviduct obstruction. These findings underscored the influence of coinfecting pathogens on the pathogenicity of *R. anatipestifer* and provide new insights for developing control strategies against this bacterial infection.

## Data Availability

The original contributions presented in the study are included in the article, further inquiries can be directed to the corresponding author.

## References

[1] Segers P Mannheim W Vancanneyt M De Brandt K Hinz KH Kersters K . *Riemerella anatipestifer* gen. Nov., comb. nov., the causative agent of septicemia anserum exsudativa, and its phylogenetic affiliation within the Flavobacterium-Cytophaga rRNA homology group. Int J Syst Bacteriol. (1993) 43:768–76. doi: 10.1099/00207713-43-4-768, 8240957

[2] García-López M Meier-Kolthoff JP Tindall BJ Gronow S Woyke T Kyrpides NC . Analysis of 1,000 type-strain genomes improves taxonomic classification of bacteroidetes. Front Microbiol. (2019) 10:2083. doi: 10.3389/fmicb.2019.02083, 31608019 PMC6767994

[3] Rubbenstroth D Hotzel H Knobloch J Teske L Rautenschlein S Ryll M. Isolation and characterization of atypical *Riemerella columbina* strains from pigeons and their differentiation from *Riemerella anatipestifer*. Vet Microbiol. (2011) 147:103–12. doi: 10.1016/j.vetmic.2010.06.008, 20615634

[4] Abd El-Hamid MI Awad NFS Hashem YM Abdel-Rahman MA Abdelaziz AM Mohammed IAA . In vitro evaluation of various antimicrobials against field *mycoplasma gallisepticum* and *mycoplasma synoviae* isolates in Egypt. Poult Sci. (2019) 98:6281–8. doi: 10.3382/ps/pez576, 31579902 PMC8913763

[5] Shousha A Awad A Younis G. Molecular characterization, virulence and antimicrobial susceptibility testing of *Riemerella anatipestifer* isolated from ducklings. Biocontrol Sci. (2021) 26:181–6. doi: 10.4265/bio.26.181, 34556621

[6] Lyu Z Han S Li J Guo Z Geng N Lyu C . Epidemiological investigation and drug resistance characteristics of *Riemerella anatipestifer* strains from large-scale duck farms in Shandong Province, China from march 2020 to march 2022. Poult Sci. (2023) 102:102759. doi: 10.1016/j.psj.2023.102759, 37209657 PMC10209456

[7] Hao J Zhang J He X Wang Y Su J Long J . Unveiling the silent threat: a comprehensive review of *Riemerella anatipestifer* - from pathogenesis to drug resistance. Poult Sci. (2025) 104:104915. doi: 10.1016/j.psj.2025.104915, 40020410 PMC11919424

[8] Omaleki L Blackall PJ Bisgaard M Turni C. Molecular and serological characterization of *Riemerella* isolates associated with poultry in Australia. Avian Pathol. (2021) 50:31–40. doi: 10.1080/03079457.2020.1828568, 32990455

[9] Ke T Yang D Yan Z Yin L Shen H Luo C . Identification and pathogenicity analysis of the pathogen causing spotted spleen in Muscovy duck. Front Vet Sci. (2022) 9:846298. doi: 10.3389/fvets.2022.846298, 35677936 PMC9169529

[10] Yang Z Yang X Wang M Jia R Chen S Liu M . Genome-wide association study reveals serovar-associated genetic loci in *Riemerella anatipestifer*. BMC Genomics. (2024) 25:57. doi: 10.1186/s12864-024-09988-4, 38216873 PMC10787497

[11] Chen Y Li X Liu Z Hu M Ma J Luo Y . Genomic analysis and experimental pathogenic characterization of *Riemerella anatipestifer* isolates from chickens in China. Poult Sci. (2024) 103:103497. doi: 10.1016/j.psj.2024.103497, 38346372 PMC10867588

[12] Lv J Chen H Ma X Cong Y Song X Li Y . Genome analysis screening virulence genes for the altered pathogenicity of *Riemerella anatipestifer* in hens. Front Microbiol. (2025) 16:1705927. doi: 10.3389/fmicb.2025.1705927, 41450938 PMC12727888

[13] Zhang C Liu D Sui Z E W Gogoi-Tiwari J Liu H . Epidemiological investigation of *Riemerella anatipestifer* in large-scale chicken farms in 29 provinces of China from 2021 to 2024. Poult Sci. (2025) 104:105467. doi: 10.1016/j.psj.2025.105467, 40602097 PMC12269571

[14] Liu J Hao D Ding X Shi M Wang Q He H . Epidemiological investigation and β-lactam antibiotic resistance of *Riemerella anatipestifer* isolates with waterfowl origination in Anhui Province, China. Poult Sci. (2024) 103:103490. doi: 10.1016/j.psj.2024.103490, 38387287 PMC10899037

[15] Zhang Y Wang X Wang Y Sun J Dong W Meng K . Biological and genomic characteristics of chicken-derived *Riemerella anatipestifer* in China. Front Microbiol. (2025) 16:1652106. doi: 10.3389/fmicb.2025.1652106, 40950598 PMC12426287

[16] Lv J Gao Y Chen H Kang M Yin D Cong Y . Emerging *Riemerella anatipestifer* infection in chickens: pathogenic characteristics and host immune response profiles. Poult Sci. (2025) 104:105687. doi: 10.1016/j.psj.2025.105687, 40840279 PMC12396462

[17] Zhou W Cui X Zhou S Liu S Peng C Yang J . Spillover of *Riemerella anatipestifer* to laying hens leads to a decrease in egg production and hatchability. Poult Sci. (2025) 104:106076. doi: 10.1016/j.psj.2025.106076, 41218555 PMC12657315

[18] Yehia N Salem HM Mahmmod Y Said D Samir M Mawgod SA . Common viral and bacterial avian respiratory infections: an updated review. Poult Sci. (2023) 102:102553. doi: 10.1016/j.psj.2023.102553, 36965253 PMC10064437

[19] Jiang L Han Z Chen Y Zhao W Sun J Zhao Y . Characterization of the complete genome, antigenicity, pathogenicity, tissue tropism, and shedding of a recombinant avian infectious bronchitis virus with a ck/CH/LJL/140901-like backbone and an S2 fragment from a 4/91-like virus. Virus Res. (2018) 244:99–109. doi: 10.1016/j.virusres.2017.11.007, 29141204 PMC7114561

[20] Zeng Z Yao L Feng H Wang Z Jiang L Wang H . Genetic and pathogenic characteristics of a novel recombinant GI-19 infectious bronchitis virus strain isolated from northeastern China. Poult Sci. (2025) 104:104985. doi: 10.1016/j.psj.2025.104985, 40081171 PMC11946749

[21] Ma T Xu L Ren M Shen J Han Z Sun J . Novel genotype of infectious bronchitis virus isolated in China. Vet Microbiol. (2019) 230:178–86. doi: 10.1016/j.vetmic.2019.01.020, 30827386 PMC7117389

[22] Mendoza-González L Marandino A Panzera Y Tomás G Williman J Techera C . Research note: high genetic diversity of infectious bronchitis virus from Mexico. Poult Sci. (2022) 101:102076. doi: 10.1016/j.psj.2022.102076, 36041394 PMC9449659

[23] Yuan W Lv T Jiang W Hou Y Wang Q Ren J . Antigenic characterization of infectious bronchitis virus in the South China during 2021-2022. Viruses. (2023) 15:1273. doi: 10.3390/v15061273, 37376573 PMC10301595

[24] Guo X Liu C Hu F Liu L Zhu T Gao Y . Dominance of the GI-19 genotype and genomic characterization of the S1 gene in avian infectious bronchitis virus from 2020 to 2024. Front Cell Infect Microbiol. (2025) 15:1640152. doi: 10.3389/fcimb.2025.1640152, 40771312 PMC12325186

[25] Lu Y Zeng Y Luo H Qiao B Meng Q Dai Z . Molecular characteristic, evolution, and pathogenicity analysis of avian infectious bronchitis virus isolates associated with QX type in China. Poult Sci. (2024) 103:104256. doi: 10.1016/j.psj.2024.104256, 39288718 PMC11421327

[26] Wu Q Xu M Wei D Zhang X Li D Mei M. Pathogenicity and molecular characterization of a GI-19 infectious bronchitis virus isolated from East China. Front Vet Sci. (2024) 11:1431172. doi: 10.3389/fvets.2024.1431172, 39170640 PMC11335494

[27] Lin SY Chen HW. Infectious bronchitis virus variants: molecular analysis and pathogenicity investigation. Int J Mol Sci. (2017) 18:2030. doi: 10.3390/ijms18102030, 28937583 PMC5666712

[28] Yan S Sun Y Huang X Jia W Xie D Zhang G. Molecular characteristics and pathogenicity analysis of QX-like avian infectious bronchitis virus isolated in China in 2017 and 2018. Poult Sci. (2019) 98:5336–41. doi: 10.3382/ps/pez351, 31222258 PMC7107289

[29] Yan K Wang X Liu Z Bo Z Zhang C Guo M . QX-type infectious bronchitis virus infection in roosters can seriously injure the reproductive system and cause sex hormone secretion disorder. Virulence. (2023) 14:2185380. doi: 10.1080/21505594.2023.2185380, 36883685 PMC10012921

[30] Stipkovits L Egyed L Palfi V Beres A Pitlik E Somogyi M . Effect of low-pathogenicity influenza virus H3N8 infection on *Mycoplasma gallisepticum* infection of chickens. Avian Pathol. (2012) 41:51–7. doi: 10.1080/03079457.2011.635635, 22845321

[31] Sid H Benachour K Rautenschlein S. Co-infection with multiple respiratory pathogens contributes to increased mortality rates in Algerian poultry flocks. Avian Dis. (2015) 59:440–6. doi: 10.1637/11063-031615-Case.1, 26478165

[32] Kuang J Xu P Shi Y Yang Y Liu P Chen S . Nephropathogenic infectious bronchitis virus infection altered the metabolome profile and immune function of the bursa of fabricius in chicken. Front Vet Sci. (2021) 7:628270. doi: 10.3389/fvets.2020.628270, 33553290 PMC7858655

[33] Huang Q Yang X Zhao X Han X Sun S Xu C . Co-infection of H9N2 subtype avian influenza virus and QX genotype live attenuated infectious bronchitis virus increase the pathogenicity in SPF chickens. Vet Microbiol. (2024) 295:110163. doi: 10.1016/j.vetmic.2024.110163, 38959807

[34] Kong L You R Zhang D Yuan Q Xiang B Liang J . Infectious bronchitis virus infection increases pathogenicity of H9N2 avian influenza virus by inducing severe inflammatory response. Front Vet Sci. (2022) 8:824179. doi: 10.3389/fvets.2021.824179, 35211536 PMC8860976

[35] Shen YX Li WW Xia J Du JT Li SY Chen W . Research note: effects of *Escherichia coli* co-infection on the protective efficacy assessment of two common infectious bronchitis vaccines. Poult Sci. (2021) 100:101324. doi: 10.1016/j.psj.2021.101324, 34358949 PMC8350523

[36] Amarasinghe A Popowich S De Silva Senapathi U Abdul-Cader MS Marshall F van der Meer F . Shell-less egg syndrome (SES) widespread in western Canadian layer operations is linked to a Massachusetts (mass) type infectious bronchitis virus (IBV) isolate. Viruses. (2018) 10:437. doi: 10.3390/v10080437, 30126175 PMC6116215

