# Supplementary material for: Coinfection with infectious bronchitis virus exacerbates the pathogenicity of Riemerella anatipestifer in chickens
Source: Front Vet Sci. 2026 Mar 17;13:1788133. doi: 10.3389/fvets.2026.1788133 (PMC13035737; doi:10.3389/fvets.2026.1788133)
Supplement: Supplementary file 1 [file Table_1.DOCX]

| **Table S1** Information of *R. anatipestifer*-positive clinical samples used in this study | | | |
| --- | --- | --- | --- |
| Region | Type | Suspicious samples | Positive samples |
| Jinan | Layers | 25 | 12 |
|  | Broilers | 21 | 7 |
|  | Layer breeders | 13 | 2 |
| Linyi | Layers | 17 | 5 |
|  | Broiler breeders | 21 | 7 |
| Weifang | Layers | 16 | 7 |
|  | Broiler breeders | 18 | 6 |
| Heze | Layers | 23 | 8 |
|  | Broilers | 9 | 3 |
|  | Broiler breeders | 7 | 2 |
| Yantai | Layer breeders | 15 | 3 |
|  | Broiler breeders | 21 | 10 |
| Binzhou | Broiler | 14 | 5 |
|  | Layer breeders | 15 | 6 |
|  | Broiler breeders | 18 | 4 |
| Jining | Layer breeders | 6 | 1 |
|  | Local breed | 8 | 1 |
| Liaocheng | Layers | 40 | 15 |
|  | Broilers | 21 | 6 |
|  | Broiler breeders | 6 | 2 |
| Zaozhuang | Layers | 25 | 9 |
|  | Broilers | 15 | 4 |
| Dezhou | Layers | 14 | 3 |
|  | Broilers | 16 | 7 |
|  | Layer breeders | 8 | 1 |
|  | Broiler breeders | 16 | 4 |
| Total | | 428 | 140 |
